# Exophytic Parietal Skin Metastases of Renal Cell Carcinoma

**DOI:** 10.1155/2013/196016

**Published:** 2013-12-26

**Authors:** Karim Kassam, Elizabeth Tiong, Ezra Nigar, Mahesh Kumar

**Affiliations:** Department of Oral & Maxillofacial Surgery, Northwick Park Hospital, The North West London Hospitals NHS Trust, London, UK

## Abstract

The common sites for metastasis of renal cell carcinoma are lung, kidney, adrenal glands, liver, and contralateral kidney. We report an unusual case of cutaneous metastasis of renal cell carcinoma in a 68-year-old woman who was treated for renal cell carcinoma with partial right nephrectomy and multikinase angiogenesis inhibitor (sunitinib) 10 years ago.

## 1. Introduction

Renal cell carcinoma (RCC) accounts for 90% of all renal tumours in adults. Approximately a third of patients with RCC develop distant metastases to lungs, liver, bones, adrenal glands, or contralateral kidney. Cutaneous metastasis accounts for 6% of metastatic RCC [1] and depicts a poor prognosis [2]. Cutaneous metastasis has been described in head and neck [3], genital [4], torso [5], and limbs [3]. This case report described an unusual cutaneous manifestation of metastatic renal cell carcinoma in a 68-year-old woman 10 years after partial right nephrectomy and treatment with multikinase angiogenesis inhibitor (sunitinib).

## 2. Case Report

A 68-year-old Caucasian woman was referred to our Oral & Maxillofacial Surgery Department from a Dermatology unit in October 2012 for assessment and removal of an exophytic lesion located on the left parietal area of the scalp.

The lesion was growing in size but, otherwise, asymptomatic. She was previously diagnosed with renal cell carcinoma.

Examination revealed a lesion of pulsatile nature with centrally raised and indurated lesion, red purplish in colour measuring approximately 4 cm in diameter. Our differential diagnoses included angioma, basal cell carcinoma, and cutaneous horn. A CT head scan shows that there is no involvement of the skull vault. Urgent blood tests were arranged which revealed hypercalcaemia (2.95 mmol/L) and anaemia (7.2 g/dL) which were highly suggestive that the lesion on the parietal scalp could be distant metastasis of renal cell carcinoma. Urgent excision of the lesion was arranged and the histopathology findings were consistent with metastatic renal cell carcinoma.

## 3. Presentation

### 3.1. Macroscopic Description (Figures 1(a) and 1(b))

Red-purplish in colour with centrally raised area measuring 2 × 1.3 cm with a well circumscribed base of 3.5 × 1.5 cm. In situ, it was solid and pulsatile in nature.

### 3.2. Histology Description

Histology revealed a focal area of ulceration on the epidermis. The dermis contained circumscribed tumour deposits (Figure 2(a)). The tumour deposit featured nests of cells with moderate amount of well-defined clear cytoplasm with round to oval nuclei. It also showed foci of vascular invasion (Figure 2(b)).

Immunochemistry showed that the tumour expressed CD10 and vimentin which are consistent with the pathological report.

## 4. Discussion

Cutaneous manifestation of RCC can indicate progression of disease or recurrence of RCC following treatment. The literature reported that cutaneous metastasis of RCC usually presents up to 5 years following initial diagnosis and after performing nephrectomy [6]. The most common cutaneous metastasis of RCC is in body sites other than the scalp [7]. It usually presents as a large pulsatile single lesion that grows rapidly in size due to the highly vascular nature of the lesion.

We report an unusual case of cutaneous metastasis of RCC 10 years following initial diagnosis and right partial nephrectomy which reflected progression of her disease. The patient presented with a pulsatile exophytic lesion which is consistent with the literature findings. Furthermore, we have identified that the lesion was superficial to the parietal branch of the temporal artery which also explains its pulsatile nature.

The mechanism of cutaneous metastasis can be due to direct extension of RCC to cutaneous tissues, lymphatic or haematogenous spread [4]. In our case, the most likely mechanism is haematogenous spread to head and neck region due to rich vascular structure of this type of tumour. The literature has also suggested tumour-related growth factors such as parathyroid-related protein which may affect the localisation of this tumour in the head and neck region [4]. In addition to treating the underlying RCC, the management of cutaneous metastatic RCC lesions is usually surgical removal but radiotherapy can be considered in carefully selected cases. The choice of treatment should be an informed shared decision made between the patient and the clinical team taking into consideration the site and nature of the lesion, patient comorbidities, and patient wishes. Our patient presented with an exophytic scalp lesion which was distressing our patient with the potential for causing bleeding should the lesion invade the temporal artery. Therefore, the shared decision made was for surgical removal of the lesion while continuing with palliative treatment of RCC.

## 5. Conclusion

Cutaneous metastatic lesions are unusual manifestation of RCC. Patients presenting with nonhealing skin lesions, especially if they have had a history of renal cell carcinoma and impaired renal function, should prompt urgent assessment as it can be a sign of undiagnosed or recurrence of RCC.

## Figures and Tables

**Figure 1:**
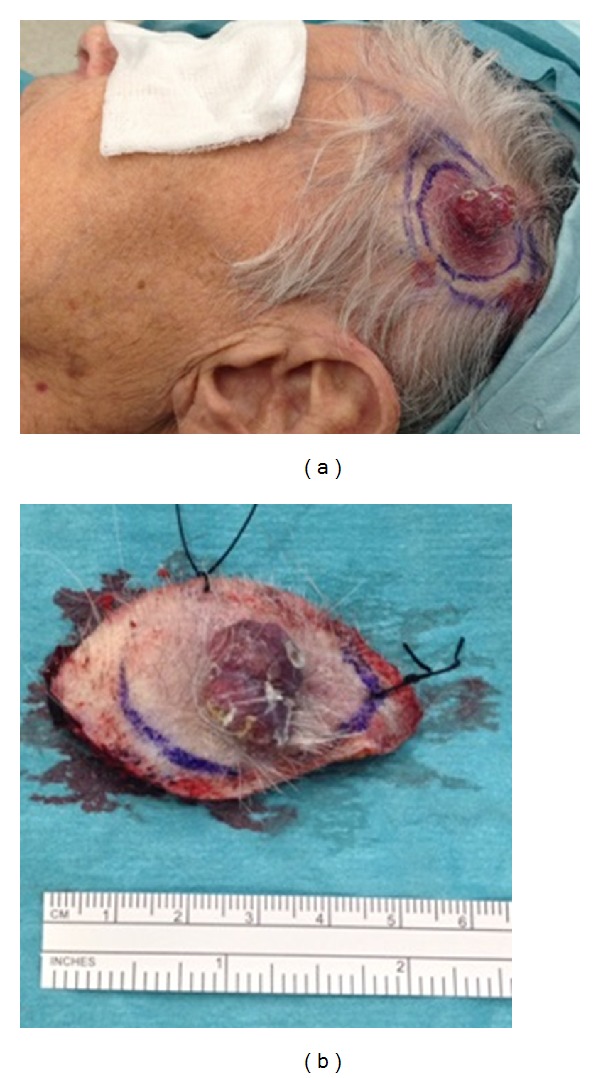


**Figure 2:**
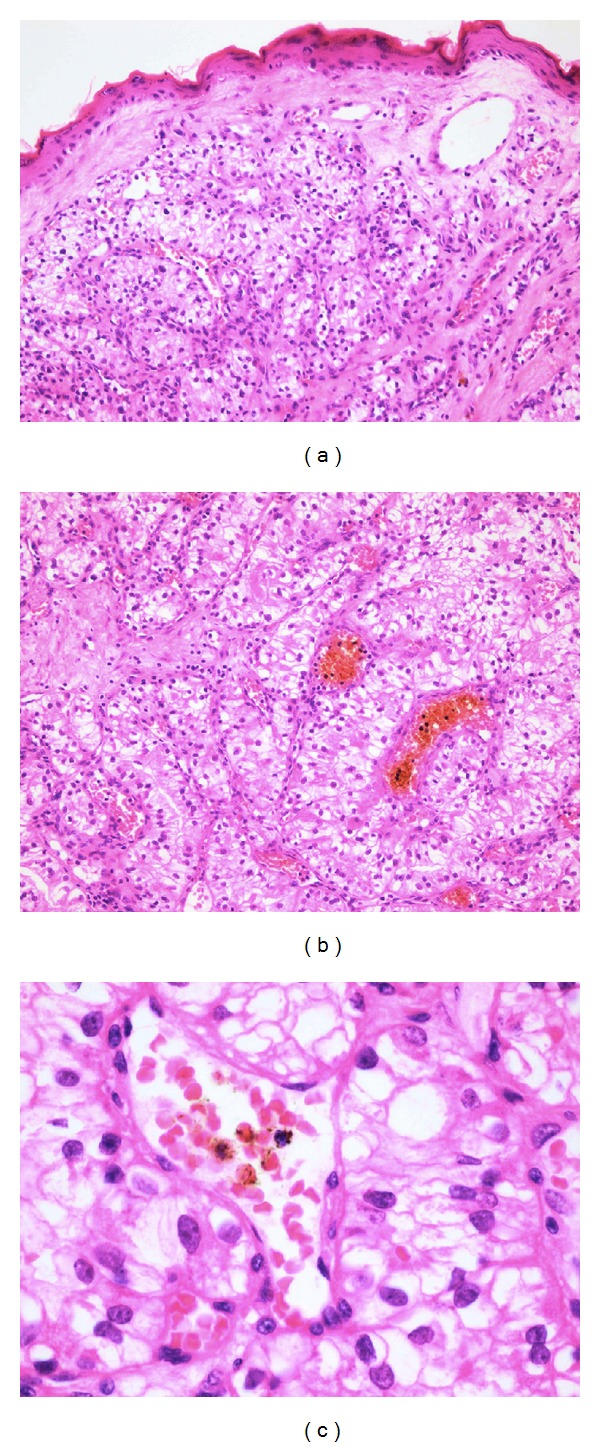
(a) ×40 magnification: the skin shows a tumour beneath the epidermis composed of clusters of clear cells and a rich vascular stroma; (b) ×100 magnification: tight nests of clear cells separated by thin richly vascular fibrous septa; (c) ×400 magnification: typical renal cell carcinoma cells with occasional prominent nucleoli.
